# Our Canada Edition

**Published:** 1898-07

**Authors:** 


### EDITORIAL.

### OUR CANADA EDITION.

THIS issue will reach more than one thousand active veterinarians in Canada and the Northwest. It will be read by more than fifteen hundred graduates of Canadian veterinary colleges, and we trust that it will in some measure add to the benefit of this entire number individually and collectively. It will, we hope, bring more closely in touch with one another this very large number of veterinarians in this rich and progressive country of North America. In some we hope it will awaken a keener interest in the veterinary journalism of America, and thus bring to one and all a greater measure of responsibility of what each should share in its work, its advancement and progress. Canadian veterinarians outside of the Province of Manitoba have not been well enough organized, and many reform measures greatly needed for the good of all have not progressed and prospered as they should. Legislation, protective and needful, has not been forthcoming, and much that should have come to the profession in the way of support and recognition has not been attained. United efforts properly directed should have brought this about long ago.

